# CAR-T cells neurotoxicity from consolidated practice in hematological malignancies to fledgling experience in CNS tumors: fill the gap

**DOI:** 10.3389/fonc.2023.1206983

**Published:** 2023-06-16

**Authors:** Lidia Gatto, Ilaria Ricciotti, Alicia Tosoni, Vincenzo Di Nunno, Stefania Bartolini, Lucia Ranieri, Enrico Franceschi

**Affiliations:** ^1^ Department of Oncology, Azienda Unità Sanitaria Locale (AUSL) Bologna, Bologna, Italy; ^2^ Department of Medical and Surgical Sciences, University of Bologna, Bologna, Italy; ^3^ Nervous System Medical Oncology Department, IRCCS Istituto delle Scienze Neurologiche di Bologna, Bologna, Italy

**Keywords:** CAR-T therapy, neurotoxicity, glioma, glioblastoma (GBM), immune effector cell-associated neurotoxicity syndrome (ICANS), ASTCT grading scale

## Abstract

Chimeric antigen receptor (CAR-T) therapy has marked a paradigm shift in the treatment of hematological malignancies and represent a promising growing field also in solid tumors. Neurotoxicity is a well‐recognized common complication of CAR-T therapy and is at the forefront of concerns for CAR-based immunotherapy widespread adoption, as it necessitates a cautious approach. The non-specific targeting of the CAR-T cells against normal tissues (on-target off-tumor toxicities) can be life-threatening; likewise, immune-mediate neurological symptoms related to CAR-T cell induced inflammation in central nervous system (CNS) must be precociously identified and recognized and possibly distinguished from non-specific symptoms deriving from the tumor itself. The mechanisms leading to ICANS (Immune effector Cell-Associated Neurotoxicity Syndrome) remain largely unknown, even if blood-brain barrier (BBB) impairment, increased levels of cytokines, as well as endothelial activation are supposed to be involved in neurotoxicity development. Glucocorticoids, anti-IL-6, anti-IL-1 agents and supportive care are frequently used to manage patients with neurotoxicity, but clear therapeutic indications, supported by high-quality evidence do not yet exist. Since CAR-T cells are under investigation in CNS tumors, including glioblastoma (GBM), understanding of the full neurotoxicity profile in brain tumors and expanding strategies aimed at limiting adverse events become imperative. Education of physicians for assessing individualized risk and providing optimal management of neurotoxicity is crucial to make CAR-T therapies safer and adoptable in clinical practice also in brain tumors.

## Introduction

Immunotherapy has emerged as a “game-changing” treatment of solid cancers, dramatically increasing survival, but the immunosuppressive characteristics of brain microenvironment have so far slowed down and hindered its success in the treatment of CNS tumors.

Currently, with the deeper knowledge of cancer-immune biology and the growing awareness that CNS is not a “sanctuary” anymore, CAR-T cell immunotherapy is attracting attention as a promising novel approach also in the field of neuro-oncology (1–5).

**Figure 1:**
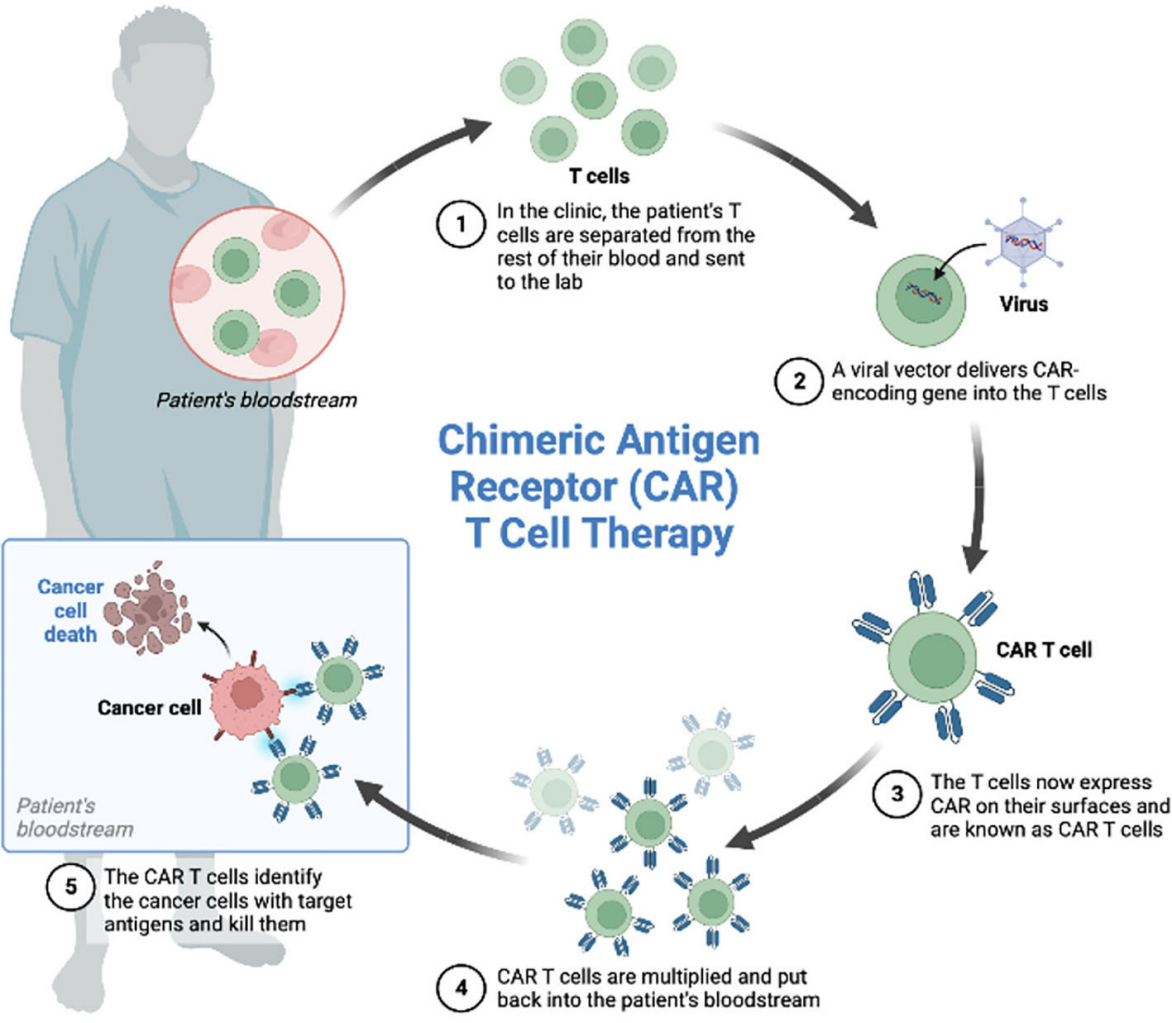
CAR-T cells manufacturing.

CAR-T cell therapy consists of collection of patient’s T cells by leukapheresis, subsequent engineered manipulation by adding a gene for a receptor (called a chimeric antigen receptor, “CAR”), ex vivo expansion and reinfusion (**Figure 1**). Each CAR is produced to target a specific cancer cell antigen (2, 6, 7).

First-generation CARs are very basic products that incorporate an antibody fragment, the single-chain variable fragment (scFv), which is the antigen-binding counterpart, recognizing a specific protein on the cancer cell’s membrane, and an intracellular signaling domain, which are connected to each other via a transmembrane domain.

Second and third generation CARs are characterized by the addition to intracellular signaling domain of one or two costimulatory domains, such as CD28 and 4-1BB, to enhance the effector activity of T lymphocytes (2) (8).

Additionally, fourth generation CARs have been recently developed. These are products capable of delivering biomolecules or cytokines (IL-12, IL-7, IL-18, IL-15), to promote antitumor activity sustained by intense inflammatory reaction and cytokine-mediated killing (9).

Currently there are six CAR T cells targeting CD19 approved by the Food and Drug Administration for the treatment of hematological cancers (Tisagenlecleucel, Axicabtagene ciloleucel, Brexucabtagene autoleucel, Lisocabtagene maraleucel, Idecabtagene vicleucel, Ciltacabtegene autoleucel); however, CAR-T cell therapies for brain tumors do not yet represent, to date, a standard treatment approved by drug regulatory entities (1).

Gliomas represent the most common primary malignant brain tumor in adults accounting for around 80% of all CNS cancers. GBM is the most common histology (almost 50% of all gliomas) and certainly the most aggressive, with a median overall survival (OS) of 13-14 months and a 5-year survival rate less than 5% (10, 11).

Despite notable advancements in CNS biology, immunology, genomics and precision medicine, neither surgery, radiation nor systemic treatments (12) can completely control high-grade gliomas. Recurrence is inevitable after conventional multimodal therapies and the prognosis remains unsatisfactory, underscoring the urgent need for novel effective therapeutic options (13, 14).

Most of the studies on CAR-Ts in CNS tumors involve adult patients with GBM, showing a modest efficacy (2, 15).

These studies, beyond the still immature efficacy data, have demonstrated the feasibility and safety of CAR-T cells for the treatment of brain tumors, increasing the interest in the application of this research in the field of neuro-oncology, also including more and more different histological subtypes (2).

Although CAR-T cells have proved to be very promising in hematologic malignancies and in solid tumors, toxicity remains a major problem due to possible immune-related adverse effects (off-tumor toxicities against the lung, the brain and the heart), even fatal.

The signature toxicity of CAR-T cell therapy is cytokine release syndrome (CRS), which typically occurs within 1-2 weeks of dosing (4, 16). Well-described in hematologic malignancies, CRS is strictly related to CAR-T cells doses and tumor burden. Patients with CRS present a variety of symptoms such as high fever, chills, fatigue, hypotension, headache, tachycardia, dyspnea, respiratory insufficiency, capillary leak and even and even life-threatening multi-organ failure.

CRS is due to due to the release of several inflammatory cytokines such as IL-6, interferon gamma (IFN-γ), IL-1, IL-2, IL-10 (4).

The second most common complication of administration of CAR-T cell is potential risk of neurotoxicity. Neurological symptoms have been recorded as ICANS (Immune effector Cell-Associated Neurotoxicity Syndrome) and include various symptoms such as headaches, confusion, agitation, seizures, tremors, trouble regarding speaking and understanding, aphasia, cranial nerve abnormalities and visual hallucination.

Other possible serious side effects of CAR T-cell therapy include allergic reactions, changes in plasma electrolyte levels (potassium, sodium or phosphorous), anemia, leukopenia and neutropenia resulting in an increased risk of infections, fatigue or bleeding.

It is essential that CAR-T treatment is conducted in highly expert centers, with multidisciplinary teams dedicated to the recognition and management of this very peculiar toxicity profile (17, 18).

Obviously, for CNS tumors, safety is paramount, as brain tissue is particularly prone to inflammation and any damage may cause long-term life-threatening sequelae (2).

Currently, also due to the very limited knowledge and the small amount of published studies, the scientific literature lacks systematic reviews and evidence-based guidelines on CAR-T toxicities in brain tumors and their clinical management. In this review, we will focus on neurotoxicity of CAR-T cell treatment in CNS tumors, discussing which conditions may favor the development of ICANS and possible strategies to limit it.

Certainly, the trials to date available in humans for CAR-T cell treatment adoption in CNS tumors are few, in a very preliminary phase and include only small casuistries: research efforts are needed to fully understand the toxicity profile of CAR-T cell therapy in brain tumors and to improve strategies aimed at limiting toxicity while maintaining efficacy unchanged (19).

## ICANS: clinics and pathogenesis

ICANS is the second most common acute toxicity observed with CAR T‐cell therapy. It, typically, occurs during CRS or more commonly after CRS decrease/resolution.

The most significant risk factor for ICANS is the presence of antecedent severe CRS and its severity is closely related to the severity of CRS, suggesting that the two phenomena are the consequence of each other, sharing, albeit partially, some pathogenic mechanisms. It is likely that the systemic inflammatory response associated with CRS underpins the development of neurotoxicity (20, 21).

Neurotoxicity typically occurs within 4-10 days of CAR-T cell infusion, but incidence rates, rate of progression and clinical presentation are variable based on the CAR-T product received.

In most cases ICANS is mildand self-limited; symptoms can include transient cognitive impairment, speech disorders and/or handwriting defects, dysgraphia, apraxia, dysgraphia and dyscalculia, or even non-specific clinical manifestations such as agitation, tremor and headache, lethargyand coma which may require the patient to be transferred to intensive care.

In most cases, the symptoms related to neurotoxicity have no radiological equivalent in MRI. Only in cases of very severe neurotoxicity, abnormal MRI imaging patterns have been described, particularly microhemorrhages, white matter changes, cerebral edema, and/or diffuse leptomeningeal enhancement. When MRI detectable changes are present, the risk of more severe neurotoxicity associated with an unfavorable outcome is greater (22–24).

Despite the clinical features of ICANS are readily recognizable, its pathophysiology is not fully elucidated (25).

Similar to CRS, the pathophysiology of ICANS seems to be related with the production of pro-inflammatory cytokines and chemokines by CAR-T cells and the activation of immune cells as macrophages in the tumor microenvironment (TME). High blood concentrations of inflammatory cytokines (such as IL-1, IL-6, IL-2, IL-15, IL-10, tumor necrosis factor (TNF-α), interferon-gamma (INF-γ), granulocyte-macrophage colony-stimulating factor and the chemokines CXCL8 and CCL2) accumulate in the blood, activate endothelial cells, alter the permeability of the BBB and spread in the brain parenchyma, activating the resident microglia (25). Activated CAR-T cells easily cross the damaged BBB reaching CSF, thus amplifying this mechanism exponentially.

Gust and colleagues (26) demonstrated that neurotoxicity pathogenesis is a cytokine-mediated phenomenon, and, at the same time, the result of endothelial activation which impairs the BBB integrity, with cerebrospinal fluid infiltration by inflammatory cytokines and leukocytes (27–30).

INF-γ and TNF induce the release of IL-6 and VEGF from pericytes, causing endothelial activation and disruption of the BBB. Following endothelial activation, von Willebrand endothelial bodies release high levels of angiopoietin-2 (Ang-2) and von Willebrand factor (VWF), resulting in coagulopathy, edema and micro/macro bleedings (26).

The main role of endothelial activation in the development of ICANS is confirmed from elevation of blood endothelial distress markers, such as EASIX score (Endothelial Activation and Stress Index) for grade ≥3 CRS and/or ICANS (26).

Angiopoietin-1 is constitutively produced by platelets and perivascular cells and has the function of stabilizing the endothelium.

Angiopoietin-2, instead, is stored in endothelial Weibel–Palade bodies and released after inflammatory stimuli, displacing ANG1 and thus increasing endothelial activation and microvascular permeability (25, 26, 28).

Significant alterations of ANG2/ANG1 ratio together with elevation of serum concentrations of VWF and chemokines have been observed in patients with severe ICANS (25, 31).

Another important element is that blood concentration of IL-6 seems to be correlated to the development of grade ≥4 neurotoxicity in the first six days after CAR-T cells infusion, with a decreasing risk in the days after. This suggest that an early onset of high concentrations of blood cytokines and a vessel disfunction are associated with neurotoxicity.

In conclusion, patients at high risk to develop severe neurotoxicity exhibit endothelial activation, high ANG2/ANG1 ratio, high blood concentrations of VWF, a tendency to coagulopathies and high blood concentration of IL-6: early identification of these risk categories to implement strategies aimed at preventing neurotoxicity is a challenge that cannot be overlooked in anticipation of a more massive adoption of CAR-t cells in clinical practice.

## ICANS in CAR-T hematological trials: clinical presentation, grading and management

ICANS secondary to CAR-T cell treatment has been well documented in hematologic malignancies (32).

JULIET phase 2 trial demonstrated the efficacy of CAR-T cell therapy Tisagenlecleucel in adult patients with diffuse large B-cell lymphoma resistant to primary and second line therapies or relapsed after stem-cell transplantation, with 40% of the patients showing complete responses and 12% partial responses. The most common grade 3 or 4 adverse events within the first 8 weeks after infusion included CRS (22%), ICANS (12%), prolonged cytopenias (32%), infections (20%) and febrile neutropenia (14%). Regarding neurotoxicity, ICANS of any grade occurred in 21% of the patients, presenting with the following symptoms: confusional state (9%), encephalopathy (6%), tremor (5%), dysphagia (4%), aphasia (3%), attention defects (3%), agitation (2%), seizure (2%), lethargy (1%), loss of consciousness (1%). About 12% of patients, instead, had grade 3 or 4 neurologic events, the majority of which resolved by supportive treatment (e.g., high dose of corticosteroids).

Similarly, in ZUMA trial (33), establishing the effectiveness Axicabtagene Ciloleucel for Refractory Large B-Cell Lymphoma, grade 3 or worse CRS and ICANS occurred respectively in 13% and 28% of the participants. Neurologic side effects occurred within 7 days from infusion and the most common events of grade 3 or worse resulted encephalopathy (21% of patients), confusional state (9%), aphasia (7%) and drowsiness (7%). Minor neurologic effects included dysphasia, attention, handwriting or calculation disturbance (27, 34).

The TRASCEND trial (35) demonstrated the effectiveness of lisocabtagene maraleucel for patients with relapsed or refractory large B-cell lymphomas, at cost of a moderate incidence of CRS and neurological events. Grade 3 or worse CRS and neurological events occurred respectively in 2% and 10% of participants (35). ICANS of any grade occurred in 30% of patients, mainly after the onset of CRS (73% of cases); the most common symptoms were encephalopathy (21% any grade, 7% grade 3-4), aphasia (10% any grade, 2% grade 3-4), delirium (6% any grade, 1% grade 3-4) and headache (3% any grade, 1% grade 3-4).

A large metanalysis (36) analyzed data from 35 hematologic cancer studies with CAR-T cell therapy, including data from JULIET, ZUMA and TRANSCEND trial, for a total of 1412 patients. The pooled proportion of severe neurotoxicity (grade 3-4) was not negligible, affecting 21.7% of 747 patients.

Factors associated with a higher risk of ≥grade 3 ICANS included: higher disease burden, low platelet counts, consumptive coagulopathy and early development of severe CRS (36).

Accurately establishing ICANS grading is critical in order to manage neurotoxicity as best as possible. There are many grading score systems to measure ICANS and the first was the CTCAE, but it was not considered optimal because it was not designed specifically for CAR-T cell therapy trials, thus, it was not enough accurate for capturing the severity, timing, and spectrum of neurotoxicity. Specifically, it leaved much more subjectivity without discerning the clinically relevant findings that define immune effector cell-mediated events from non-specific ones (22, 37).

The American Society for Transplantation and Cellular Therapy (ASTCT) has proposed the ASTCT Consensus Grading for Cytokine Release Syndrome and Neurologic Toxicity Associated with Immune Effector Cells in order to standardize CRS and ICANS grading (37, 38).

The ASTCT grading scale for ICANS uses a tool called the Immune Effector Cell-Associated Encephalopathy (ICE) score.

ICE is a score based on five parameters: *orientation* (year, months, city, hospital), *attention* (ability to count backwards from 100 to 10), *naming* (ability to name three simple objects), *ability to follow simple commands*, and *handwriting* (ability to write a standard sentence). Each parameter of ICE score gives points to the patient and it is used to determine the grading of ICANS.

ICE score is determined on all CAR-T cell therapy patients at baseline, after infusion, and is administered a minimum of twice daily or more frequently if ICANS is suspected.

ICANS’ grading is determined by combining ICE score with other parameters, including conscious level, presence of seizures, motor disturbance and presence of cerebral edema.

A patient with aphasia, even if ICE 0, is considered ICANS 3 if he is awakenable and ICANS 4 if he is not awakenable. Cerebral hemorrhage, with or without associated edema, is not considered a manifestation of neurotoxicity and so is not used to determine ICANS grading.

ICANS treatment has evolved over time to prevent progressive neurotoxicity and to restore neurologic function (**Table 1**) (39).

**Table 1:** Icans management.

ICANS Grade	Standard of care	Steroids	Follow-up
ICANS Grade 1	Supportive care	no	•Close observation •Consider levetiracetam as prophylaxis against seizures •Consider neurologist consultancy •Consider tocilizumab in case of concurrent CRS
ICANS Grade 2	Resuscitation consultancy	10–20 mg intravenous dexamethasone every 6 hours	•If returns to grade 1 ICANS: steroids tapering in 3-4 days •If it doesn’t return to grade 1 ICANS, consider transferring patient to intensive care unit
ICANS Grade 3	Intensive Care	10–20 mg intravenous dexamethasone every 6 hours	•Consider monitoring intracranial pressure by funduscopy •Consider monitoring neuroimaging by CT or MRI every 2–3 days •If ICANS returns to grade 1: steroids tapering in 3-4 days •If ICANS doesn’t return to grade 1: intensive care transferring
ICANS Grade 4	Intensive Care	1 g intravenous methylprednisolone for at lebull 3 days, then taper at 250 mg every 12 hours for 2 days, then 125 mg every 12 hours for 2 days, then 60 mg every 12 hours for 2 days	•Patients with reduced consciousness may need intubation •In case of high intracranial pressure, consider acetazolamide and mannitol therapy

For grade 1 ICANS, management is supportive. For grade ≥2 ICANS, glucocorticoid therapy should be considered. Suggested doses include 10–20 mg intravenous dexamethasone every 6 hours for grades 2–3 ICANS and 1 g intravenous methylprednisolone for at least 3 days for grade 4 ICANS (27, 30, 40).

Patients with ICANS grade 3 or worse, should ideally be managed in an intensive care setting. Treatment consists of glucocorticoids, with or without immunomodulatory drugs, as siltuximab, an anti-IL-6 antibody (41) and the IL-1 receptor antagonist, anakinra (**Table 1**). However, the role of anti-IL-6 and anti-IL-1 agents and the precise indication for their use remain to be determined.

It is crucial to early identify neurotoxicity at symptom onset, as it requires experience as well as appropriate and immediate treatments.

## ICANS in neuro-oncology

The potential for neurotoxicity, considering the sensitivity of the brain tissue to inflammation and the risk of severe localized inflammation in the enclosed intracranial space, is still at the forefront of concerns for CAR-T cell administration in CNS tumors (19, 41, 42).

As we learned from hematological malignancies, CRS and ICANS onset is strictly related to the high tumor burden and high antigen load, but patients with brain malignancies do not bear large tumor burden, and, thus, are not expected to develop severe systemic CRS or severe off-tumor neurotoxicity (43).

The efficacy and safety of CAR-T cell therapy in glioma patients have been investigated in several phase I/II studies, showing a wide range of response rates (15, 44, 45). However, the most interesting aspect that emerges is that CAR-T neurotoxicity, especially systemic, paradoxically, is much lower in brain tumors than in hematological malignancies and, globally, to date, CAR T-cell trials in glioma patients have not shown severe CRS and ICANS (15, 44, 45). CAR-T cell therapy seems a relatively safe therapeutic option in gliomas and neurotoxicity has been shown to be limited and manageable.

More than the risk of generalized systemic off-tumor on target toxicity, in gliomas subjected to CAR-T therapy, local neurological toxicity has been observed, mainly due to intracranial compartmentalized cytokine release within the tumor mass, a condition known as tumor inflammation-associated neurotoxicity (TIAN) (46). This is partially explained by the local administration of CAR-T cells, preferred for brain tumors, which allows to increase the effectiveness and, at the same time, to limit the generalized systemic toxicity.

Conversely, the systemic intravenous administration of CAR-T cells in glioma, besides ineffective, is also associated with major toxicity, especially of the lung type, in one case even fatal (45).

One of the major challenges in CAR-T therapy of gliomas is the identification of tumor specific antigens that may be ideal, i.e. expressed by most tumor cells, but absent in other organs and healthy brain tissue, thus reducing the potential for toxicity of immunotherapy. Promising cell surface markers that have been attempted in clinical trials as potential target antigens for CAR-T cell therapy to treat gliomas include: IL13Rα2, GD2, HER2, EphA2 and EGFRvIII (15, 46, 47).

Due to the high specificity of interleukin-13 receptor alpha 2 (IL13Rα2) for GBM with scarce expression in the surrounding normal brain tissue, IL13Ra2 was the first target of CAR-T cell therapy in GBM.

In the first-in-human clinical trial NCT00730613, Brown et al. (48) experienced direct IL13Rα2 CAR-T cells infusion into the post-surgical resection cavity of three patients diagnosed with recurrent GBM. They performed up to 12 “adjuvant” infusions of IL13Rα2 CAR-T cells at a maximum dose of 108 cells: excellent antitumor response in two of the three treated patients was observed and only mild side effects such as headaches and transient non-localizing neurologic deficits were described (48) (1). In particular, one patient reported a 7.5-month regression period with 11 months of median OS. At the 108 T cell dose, only grade 3 headache occurred in one patient. Another patient, who had the highest level of IL13Rα2 expression, experienced a very brief and reversible episode of gait instability and tongue deviation, possibly attributed to T cell administration. This neurological event occurred after the twelfth and final CAR T cell infusion, required hospital admission, and was treated with low-dose single administration of dexamethasone.

To prove IL13Rα2-directed CAR-T cells efficacy and trafficking, another trial of IL13Ra2 CAR-T cells incorporating positron emission tomography (PET) imaging was performed, demonstrating adequate CAR T cells trafficking into brain parenchyma (49).

Furthermore, notably, in another study, intracavitary infusion of IL13Rα2-directed CAR-T cells followed by additional doses of intracerebroventricular infusion via lateral ventricles caused a noteworthy objective intracranial and spinal response in a patient at a late stage of multifocal GBM with a persistent clinical response for 7.5 months (1, 15). The general toxicity and neurotoxicity were negligible, despite a significant increase in inflammatory cytokines and chemokines levels, such as CXCL9 and CXCL10 (15).

Approximately 40% of GBMs harbor EGFR amplification or constitutively express EGFRvIII. EGFRvIII is selectively expressed only on tumor tissue and is not found in healthy tissues, so EGFRvIII has been assessed as an attractive target.

In 2017, O’Rourke et al. (43) published the first-in-human clinical trial experimenting intravenously EGFRvIII-directed CAR-T cells administered to 10 GBM patients. The study revealed very limited efficacy, and clinically significant neurologic events were observed in two patients suggesting off-tumor toxicity of the systemic intravenous administration of CAR-T cells (1, 43). One subject had a seizure followed by cognitive disturbance, treated with high-dose corticosteroids, antiepileptics and siltuximab. A second patient had neuro-cognitive decline and was treated with high-dose corticosteroids followed by siltuximab, but the overall clinical assessment was more consistent with disease progression. In summary, there were no dose-limiting toxicities but neurologic adverse events mainly related to localized intracranial inflammation substained by T cell activation and cytokine release.

Another clinical trial on EGFRvIII-targeted CAR-T cell therapy was published by Goff et al. (45). Third-generation EGFRvIII CAR T cells were intravenously administered after chemotherapy-induced lymphodepletion and IL-2 injections to 18 recurrent GBM patients. In the face of poor therapeutic success (median PFS was 1.3 months with no objective responses), a patient died and two patients developed dose-dependent T cell-induced acute respiratory distress secondary to pulmonary edema (45). In particular, one patient developed acute dyspnea and hypotension which required intensive care intervention but, nevertheless, the patient died within few hours. The second patient received a total dose of 3×1010 cells and developed respiratory distress, successfully treated with continuous positive airway pressure (CPAP) with complete resolution of symptoms. Thus, once again, systemic intravenous administration of CAR-T cells demonstrated limited efficacy and off-tumor toxicity (45).

Similarly, also trials on other cancer entities unveiled neurologic adverse effects associated with the systemic intravenous administration of CAR-T cells (1, 27, 50).

Durgin et al. (51) reported the case of an adult GBM patient treated with a single systemic infusion of EGFRvIII targeted CAR-T cells, who survived 36 months, far exceeding life expectancy for recurrent GBM. In this case toxicity resulted mild: the patients only reported flu-like symptoms, arthralgia, myalgia, and headache, managed conservatively with acetaminophen.

Approximately 40-50% of GBMs overexpress human epidermal growth factor receptor 2 (HER2) (4, 52). The major concern of HER2 CAR-T cell is safety issue because although HER2 is overexpressed in numerous malignancies, it can be also be expressed in healthy tissues, leading to high risk of on-target off-tumor toxicity (18).

In fact, the first reported use of HER2 CAR T cell therapy in metastatic colon cancer resulted in the death of one patient because of acute dyspnea followed by respiratory failure (18): since then, efforts have been made to improve safety associated with HER2 CAR-T cell therapy. In a phase I clinical trial up to 1 x 108 second-generation HER2 CAR-T cells were intravenously administered to 17 GBM patients: promising efficacy was shown, and the treatment was well tolerated without dose-limiting toxic effects (53). Patients received one or more intravenous infusions of autologous HER2-CAR-Ts at 5 dose levels, with 6 patients receiving multiple infusions. No dose-limiting toxicity was observed; only two patients experienced grade 2 seizures and/or headaches, probably related to the CAR-T cell infusion.

Recently, a phase I-II trial evaluating safety and antitumor activity of new generation GD2-directed CAR T cells expressing the inducible caspase 9 suicide gene in relapsed or recurrent neuroblastoma has been published (54). GD2 is a disialoganglioside antigen that is expressed on tumors of neuroectodermal origin, including neuroblastoma and melanoma (55).

The treatment resulted feasible and safe with an overall response rate of 63%. No dose-limiting toxic effects were described and 75% of enrolled patients experienced only mild CRS. The activation of the suicide gene to control side effects was required only in one patient (54).

A case-report has also been published concerning the use of CAR-T cells in a rare tumor of the CNS, the recurrent malignant meningioma (56). Malignant meningiomas are characterized by poor prognosis, rapid tumor growth and a high recurrence rate, also after multiple surgical resections and radiotherapy treatments (57). In this first-in-human study, investigators examined safety and anti-tumor activity of B7-H3-targeted CAR-T cells, administered into the tumor resection cavity by Ommaya. The treatment resulted safe, with no adverse events of grade 3 or higher. B7-H3-targeted CAR-T cells demonstrated local anti-tumor activity, with significant decrease of B7-H3 expression near the region of CAR-T-cell administration. This was also reflected in the neuro-imaging data, with MRI indicating disease stability near the Ommaya device and disease progression in tumoral sites distant from the CAR-T delivery (58).

In general, we can conclude that CAR-T systemic neurotoxicity in brain tumors is limited, compared to hematologic malignancies, due to several factors: first, minor tumor burden. Manifestation of systemic off-tumor toxicity in GBM patients is not expected because GBM patients does not bear large tumor burden. However, given the potential for the occurrence of catastrophic localized inflammation, and the enclosed intracranial space, careful attention should be given to the possibility of localized inflammation in any patient who develop new neurologic symptoms in the first month after CAR-T cell infusion.

Second, the locoregional delivery of CAR-T cells in gliomas. Limited investigations are available for brain tumors but, reiteratively, the local intracranial CAR-T administration route seems to be the better option to decrease systemic toxicity and bypass the BBB (2, 47).

In considering local administration, however, the problem of tumor inflammation-associated neurotoxicity should not be underestimated, as it requires experience as well as appropriate and immediate treatments.

To our knowledge, there are currently no direct comparison studies between local and systemic administration of CAR-T cells with the same biological target.

Direct comparison clinical trials in terms of both efficacy and tolerability are needed to better elucidate CAR-T cell delivery mechanisms in gliomas and to offer the best treatment option to patients (1).

Developing strategies to limit local intracerebral inflammation represents the main road to develop modern immunotherapy in neuro-oncology. Optimal and prompt management of symptoms is critical; therefore, CAR-T therapy should be administered in reference centers and patients should be monitored continuously, with daily reassessment, a minimum of twice daily, for early detection of any symptoms or signs of ICANS (40).

A viable alternative approach under development aimed at overcoming unmet needs associated with CAR-T cell therapy is represented by CAR constructs engineered with natural killer cells (CAR-NK cells) (59).

CAR-NKs have been proposed as promising candidates for adoptive cell therapy, because more advantageous, less toxic and more manageable when compared to CAR-Ts.

Unlike T lymphocytes, NK cells are a group of cytotoxic lymphocytes of the innate immune system exerting specific cytotoxicity activity directly recognizing target cells, without antigen presentation on major histocompatibility complex (MHC).

Their activation is completely independent from the MHC: therefore, while CAR-T therapy necessarily requires autologous T lymphocytes, often depleted by previous chemotherapy treatments, CAR-NKs make allogeneic therapy feasible. CAR-NKs represent a “off-the-shelf” ready-to-use product, that can be produced on a large scale and used on all patients at any time.

Allogeneic CAR-NKs reduce the risk of graft versus host disease (GVHD); in addition, also considering the autologous setting, CAR NK-s are safer and have lower risk of CRS and ICANS. In fact, while CAR-T cell activation is associated with massive release of inflammatory cytokines, such as IL-1, IL-2, IL-6, IL-10, IL-15 and tumor necrosis factor alpha (strictly associated with the development of CRS and ICANS), CAR NK cells release different type of cytokines, such as IFN-gamma and granulocyte-macrophage colony-stimulating factor (GM-CSF) (59). The different cytokine release profile might explain the different toxicity spectrum between CAR-Ts and CAR-NKs (60) (59) (61).

## TIAN in neuro-oncology

**Figure 2:**
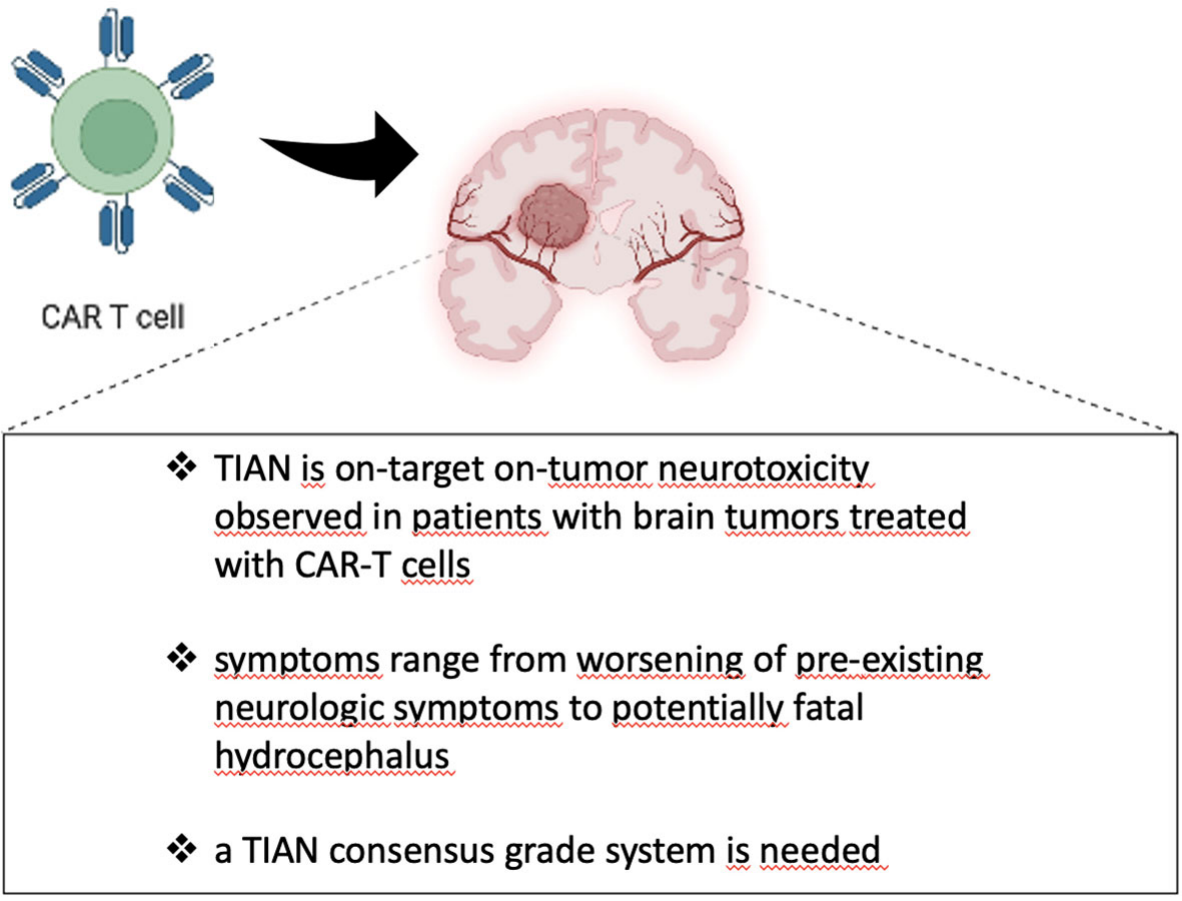
Tumor inflammation-associated neurotoxicity (TIAN).

TIAN (tumor inflammation-associated neurotoxicity) is an on-tumor, on-target neuro-toxicity syndrome, distinct from ICANS, observed in CNS tumors treated with CAR-T cell therapies (**Figure 2**) (62). Its symptom spectrum varies from headache or fever to fatal hydrocephalus. The severity of symptoms is determined by several factors, including the neuroanatomical location of the tumor and the specific CAR-T target.

TIAN is the result of a local neuronal dysfunction secondary to brain inflammation that may lead to massive increase of intracranial pressure with life-threatening hydrocephalus and high risk for herniation.

ICANS and TIAN, although both neurological syndromes secondary to immunotherapy, have different pathogenetic mechanisms and have different clinical manifestations: while ICANS is a global neurological dysfunction leading to seizures, decreased level of consciousness or speaking/movement disorders, TIAN manifests with specific regional symptoms, linked to the site of the tumor and to local inflammation, without signs of widespread neuronal suffering.

TIAN was first described in a preclinical study in mice affected by H3K27M diffuse intrinsic pontine gliomas (DIPGs) and treated with GD2 CAR-T cell therapy. Interestingly, the authors described a localized brainstem inflammation in mice leading to fatal obstructive hydrocephalus (62).

The experience gained in this preclinical study was useful to the authors in adopting and developing appropriate safety measures (for example Ommaya device) in the subsequent first-in-human phase I clinical trial with GD2-directed CAR-T cells applied intracerebroventricularly in patients diagnosed with H3K27M-mutated DIPG or spinal cord diffuse midline glioma (DMG) (46).

Within TIAN’s clinical manifestations, it is possible to identify two different syndromes, type 1 TIAN and type 2 TIAN (62). Type 1 TIAN reflects peritumoral therapy-related inflammation and is the result of increased intracranial pressure associated with obstruction of CSF flow which can potentially lead to herniation (for example, subfalcine herniation, uncal herniation or tonsillar herniation, depending on the location of the tumor). Type 2 TIAN consists of a neuronal dysfunction related to local inflammation without CSF obstruction/hydrocephalus and, clinically, it manifests with a worsening of pre-existing neurological symptoms.

While severe type 1 TIAN is considered a neurological emergency, type 2 often requires more conservative treatment, with supportive care only.

In the phase I clinical trial by Majzner and colleagues (46) several neurological symptoms consistent with TIAN in sites of CNS disease were observed; conversely, no systemic CAR-T cell on-target off-tumor toxicities were observed (1) (46). Because of high risk of cerebral edema and increased intracranial pressure, important precautions were taken, as CSF drainage via Ommaya device. Interestingly, radiographic and clinical response was observed in three out of four patients after a single dose of 1 × 106/kg GD2-CAR-T cells. One patient experienced an acute episode of fever, hypertension, decreased responsiveness and hemiplegia treated first with tolicizumab and then with Anakinra (an IL-1R antagonist). In addition, Ommaya device was accessed for CSF drainage, and the symptoms subsided quickly. Another patient underwent worsened trismus limiting oral intake to eating. He was treated with tocilizumab and anakinra, but no corticosteroids, with significant improvement in trismus. After the second dose of 30 × 106 GD2-CAR-T cells administered intra-cerebroventricularly the patient developed hydrocephalus. The Ommaya was accessed for CSF drainage and hydrocephalus resolved after 2 days. Patient 3 developed transiently increased ataxia successfully treated with tocilizumab and anakinra, but no glucocorticoids. Patient 4 exhibited encephalopathy. Due to the large disease burden, the authors were unable to distinguish between ICANS or, alternatively, a clinical manifestation of the tumor itself. The patient was successfully treated with high-dose corticosteroids, anakinra and siltuximab with resolution of encephalopathy within a few days (46).

In conclusion, the differential diagnosis between ICANS and TIAN is fundamental, as the therapeutic approach to the two syndromes is completely different. Identifying patients at high risk of developing hydrocephalus during cellular immunotherapy and adopting adequate precautionary measures are important objectives to ensure safety of these treatment in CNS tumors.

## Strategies to decrease neurotoxicity of CAR-T cell therapy

Strategies to avoid the release of large amounts of proinflammatory cytokines

and to decrease CAR-T cell toxicity are under investigation. These include (I) administration of high dose corticosteroids, (II) locoregional administration of CAR-Ts to avoid systemic toxicities, (III) agents targeting the IL-6 receptor, (IV) agents targeting the IL-1 receptor, (V) CAR products expressing suicide switch molecules, ((VI) focused ultrasound (FUS), (VII) autonomous self-regulated CAR-T cells (63).

CAR-T cell therapy-related toxicity could lead to life-threating side effects and bankruptcy outcome in CNS tumors, thus the development of new methods properly balancing between the need of CAR-T cell products with good expansion and persistence and the requirement of careful toxicity control is critical (2) (40).

Treatment with glucocorticoids in association with supportive management represents the standard of care, despite the possibility that corticosteroids reduce the effectiveness of CAR-T cells remains a critical concern (64–67).

A few reports have demonstrated favorable outcome in steroid-refractory ICANS treated with intrathecal methotrexate (12 mg) and hydrocortisone (50 mg) (64, 66). Asawa et al. reported clinical improvement and rapid resolution of ICANS in two patients treated with intrathecal administration of methotrexate and hydrocortisone, both refractory to previous therapy with dexamethasone and tocilizumab (66). Similarly, Shah et al. have reported two cases of steroid-refractory ICANS successfully treated with intrathecal combination of methotrexate (12 mg) + hydrocortisone (50 mg) with no long-term complications (64).

Local delivery of CAR-T cells minimizes the risk of systemic toxicities and seems to be more advantageous in terms of therapeutic effectiveness and clinical benefit.

For brain tumors locoregional delivery strategies include intraventricular and/or intra-tumoral administration: the first modality foresees that CAR-T cells are delivered into the cerebrospinal fluid *via* the ventricular system; the intratumor modality, instead, consists of direct administration of CAR-T cells into the tumor or resected tumor cavity; both strategies require the surgical implantation of a catheter delivery device/reservoir (68) (69, 70).

Corticosteroids are relatively effective in suppressing off-tumor toxicities but they reduce the efficacy of CAR-Ts (63, 71).

Tocilizumab, an inhibitor of inteleukin-6, an inflammatory molecule involved in CRS, can be a valid option given that it is able to turn off the strong inflammation.

Although tocilizumab has an established role for the treatment of systemic CRS (43), it is not clear whether it is able to cross the BBB, reducing IL-6 levels also in brain tissue. The mechanism of action of tocilizumab, which binds the IL-6 receptor, leads to higher blood levels of IL-6, potentially increasing CNS exposure to IL-6 (72).

Siltuximab, on the other hand, is a small molecule capable of directly binding circulating IL-6 rather than the IL-6 receptor and therefore it is believed to be the agent of choice to control CRS and ICANS, preserving brain tissue from damages resulting from high levels of inflammation (41). Another no less important factor, the size of the siltuximab molecule, much smaller, allows it to cross the BBB more easily (40).

Anakinra is a recombinant version, produced with molecular biology techniques, of the human IL-1 receptor antagonist protein.

Anakinra has been shown clinical benefit in CRS and ICANS in animal models (73, 40) but also in some clinical trials conducted on humans, proving to be effective even in cases of severe toxicity resistant to tolicizumab and siltuximab (46).

However, to date, evidence supporting routine use of Anakinra is scarce and further clinical trials are urgently required.

Preclinical and translational research is developing new methods aimed at reducing the toxicity of CAR-T therapies, as an example novel models of CAR-Ts expressing suicide switch molecules or techniques for remodulating the structure of CAR-T cells by reducing their affinity for the antigen (2).

The extraordinary characteristic of these new models is the expression of a sort of “suicide gene” which works as an “on-off” switch that can be activated only in case of life-threatening toxicity and that is capable of irreversibly blocking the action of CAR-Ts.

Some strategies have already been tested in clinical trials in CNS tumors, such as the CAR-T expressing the gene coding for inducible caspase-9 (iCasp9). In case of life-threatening toxicity, iCasp9

is activated and works by eliminating CAR-T cells immediately. Phase I trials using CD2 CAR-T iCasp9 technology are ongoing in several indications, including DIPG and spinal DMG (2, 74).

Another novelty in the field of inducible CAR-T cells is the design of the FUS-CAR-T-cell therapy technology, a class of CAR-T that can be acoustogenetically and directly controlled by FUS, increasing the spatiotemporal selectivity of the of CAR-T action and reducing toxicity (63). MRI-guided FUS stimulation enables the delivery of thermal energy selectively at confined tumor regions with high resolutions, activating the CAR-T cells at the desired time and location in order to improve the safety of CAR-T therapy (63).

Finally, another method designed for controlling toxicity is the development of autonomous CAR-T cells that are self-regulated and can decide whether to initiate or withhold cytotoxic killing of target cells through a system capable of identifying whether the inflammatory cytokines in the tumor microenvironment have reached high levels (75). Autonomous CARs respond to heightened levels of inflammatory cytokines by inhibiting the cytotoxic action of the engineered T lymphocytes, thus reducing toxicities and ensuring greater safety for CAR-T therapy (75).

Many of these new strategies have only been tested in preclinical models, as an example in humanized mouse models and/or patient-derived xenografts, but translational research has important limitations because the tumor microenvironment of animals does not precisely recapitulate the human one and therefore it is not able to accurately predict the potential on-target off-tumor effect, CRS, and neurotoxicity that will then develop in humans. Adequate preclinical models capable of better recapitulate the negative adverse effects of T-cell-based therapy in humans should be developed (2, 76–78).

## Conclusions

The need to increase CAR-T cell products expansion, function and persistence to deliver robust clinical responses must be balanced with the potential risks of heightened toxicity associated with rapidly expanding CAR-T cell populations *in vivo*.

After CRS, ICANS is the second most frequent side effect observed in CAR-T therapy.

Surprisingly, unlike CAR-T cell therapy in hematological malignancy, CRS and ICANS are not common events in CNS tumors: mild/moderate self-limited neurotoxicity is observed, with great difficulty for the clinician to establish whether they are due to the action of the CAR-Ts or to the tumor itself.

Further research effort is needed to fully understand the toxicity profile of CAR-T cell therapy in brain tumors and to improve strategies aimed at limiting adverse events while maintaining efficacy unchanged (19).

Furthermore, it is important to identify patients at high risk of developing ICANS so that they can be appropriately monitored for its emergence and swiftly managed in an attempt to prevent escalation to more severe phenotypes. Improving the expertise of physicians involved in the treatment of these complex patients is the key for a safe management of CAR-T cells in in clinical practice.

## Author contributions

Writing and conceptualization: LG Supervision: EF All authors have revised the final version of the manuscript. All authors contributed to the article and approved the submitted version.
